# Rehabilitation Intervention for an Infant with Simple Epidermolysis Bullosa from NICU to Home Discharge: A Case Report

**DOI:** 10.3390/jcm14228012

**Published:** 2025-11-12

**Authors:** Tetsuo Sakai, Syoichi Tashiro, Aki Karasuyama, Toshihiko Kimura, Masami Narita, Shin Yamada

**Affiliations:** 1 Department of Rehabilitation, Kyorin University Hospital, Mitaka 181-8611, Japan; t-sakai@ks.kyorin-u.ac.jp; 2 Department of Rehabilitation Medicine, Kyorin University School of Medicine, Mitaka 181-8611, Japan; 3 Department of Rehabilitation, Kyorin University Faculty of Health Sciences, Mitaka 181-8612, Japan; aki-karasuyama@ks.kyorin-u.ac.jp; 4 Department of Pediatrics, Kyorin University School of Medicine, Mitaka 181-8611, Japan

**Keywords:** neonatal rehabilitation, habilitation, growth and development, basal membrane, respiratory rehabilitation, skin diseases, genetic

## Abstract

**Background/Objectives**: Reports detailing rehabilitative interventions for infants with severe dermatologic disorders are scarce. Epidermolysis Bullosa (EB) is a genetic disorder characterized by skin fragility, which causes blistering after minor trauma. Since there is still no cure in general clinics, symptomatic treatment and developmental support are essential for managing the condition. While physiotherapy and occupational therapy guidelines for EB exist, descriptions of neonatal habilitation/rehabilitation are insufficient. **Case:** This case report describes the longitudinal habilitation/rehabilitation intervention process for a newborn with Dowling–Meara EB, the most severe form, from admission to the Neonatal Intensive Care Unit (NICU) until discharge. Since maneuvers requiring contact were strictly limited due to skin vulnerability, rehabilitation interventions were implemented utilizing the opportunity afforded by necessary care. Intervention strategies were modified according to developmental stages and skin stability, with a particular emphasis on sensory development, postural control training, and fostering the mother–child relationship. This report is the first to describe the applicability of sensory rehabilitation and the use of behavioral cues to facilitate voluntary movements. In addition, careful respiratory rehabilitation was implemented for comorbid tracheomalacia with specific attention to skin vulnerability. The child achieved stable head/neck control, symmetrical limb movements, reaching, guided rolling, and stable oxygenation by the time of discharge. **Conclusions:** Balancing skin disorder prevention and motor–neural development requires flexible approaches that minimize contact while utilizing routine care as a training opportunity. Our experience will contribute to the progress in the habilitation, wound rehabilitation and respiratory rehabilitation of infants with severe dermatologic disorders.

## 1. Introduction

Epidermolysis Bullosa (EB) is a disease caused by genetic abnormalities in proteins involved in epidermal–dermal adhesion. This results in the separation of the epidermis and dermis with minimal force, leading to blister and erosion formation. The reported prevalence per million population is 11 in the United States [1], 12.7 in France [2], 10 in Australia [3], 15–35 in the United Kingdom [4], and 28 in Japan [5]. EB is classified into four main types: simplex, junctional, dystrophic, and miscellaneous. EB simplex accounts for approximately 40% of all EB cases [1]. The primary subtype, the Dowling–Meara type, presents the most severe clinical symptoms, with blister formation throughout the body in response to minor mechanical stimuli. Despite recent clinical progress proposing various promising treatments, such as Oleogel-S10 [6], beremagene geperpavec [7], prademagene zamikeracel [8], and Dupilumab [9,10], management still centers on symptomatic treatment aimed at preventing skin damage and secondary infections [11]. The need for rest to avoid mechanical irritation of the epidermis can, however, hinder the development of infants and young children. While physiotherapy and occupational therapy guidelines for EB have been proposed [12,13], the former is more focused on gross motor skills, which align well with the primary goal of habilitation in the Neonatal Intensive Care Unit (NICU). The physiotherapy guideline emphasizes the importance of multidisciplinary team care, incorporating early initiation to preserve muscle power and Range of Motion (ROM), and to promote development, including weight-bearing activity and independent function (recommendation level D) [13]. These elements are also crucial in preventing considerable cognitive deficits [14]. Furthermore, gentle handling is recommended as an indispensable maneuver to prevent new blister formation (recommendation level D) [13].

However, descriptions for infant patients are generally scarce, reflecting a shortage of reports on clinical practice, especially for cases presenting with the most severe symptoms and treated in the NICU. More concretely, the authors of the physiotherapy guideline have pointed out the lack of studies regarding the typical developmental achievement of infants with EB and studies on gross motor skill attainment and prone positioning to avoid the shortening and adherence of soft tissues [13].

Here, we report our experience with a Dowling–Meara-type infant who presented with the most severe skin symptoms and received rehabilitation interventions for skin lesion prevention, developmental support, and secondary impairment management starting from admission to the NICU. While the interventions include general approaches, describing the overall features of the habilitation/rehabilitation intervention is important to clarify which interventions were specific to this case or which were prioritized.

## 2. Case Presentation

### 2.1. Clinical Course Before the Initiation of Habilitation/Rehabilitation (Postnatal Days 0–81)

The patient was a male infant born at 37 weeks and 6 days of gestation via scheduled Cesarean section at a referring hospital due to breech presentation. Birth weight was 2191 g, and Apgar scores were 8 and 9 (at 1 and 5 min, respectively). Due to extensive epidermal detachment over the entire skin surface at birth, the patient was transferred to our hospital and admitted to the Neonatal Intensive Care Unit (NICU). He presented with relative hypoproteinemia due to skin exudation and received 43 whole-blood transfusions (20 albumin preparations over 30 days, six globulin preparations over 6 days, and seven red cell concentrates over 9 days). Although the respiratory status was maintained on room air until 45 days of age, oxygenation subsequently declined due to tracheomalacia, necessitating the delivery of supplemental oxygen to the mouth.

Positional changes were strictly prohibited, and the infant was kept in a supine position at all times, which made developmental positioning maneuvers challenging. Furthermore, owing to the COVID-19 pandemic, parental visits were partially restricted to one hour per day, three days per week. The mother expressed frustration at not being permitted to touch the infant, even during vesicle care.

From postnatal day 65, restrictions were eased: visitation time was extended to three hours on the three treatment days per week and two hours on the other days. Additionally, the mother was permitted to participate in vesicle care performed by the physician, though carrying, including the Cradle carry technique, remained disallowed.

### 2.2. Rehabilitation Initiation with Minimal Contact (Phase I: Postnatal Days 81–107)

**Figure 1:**
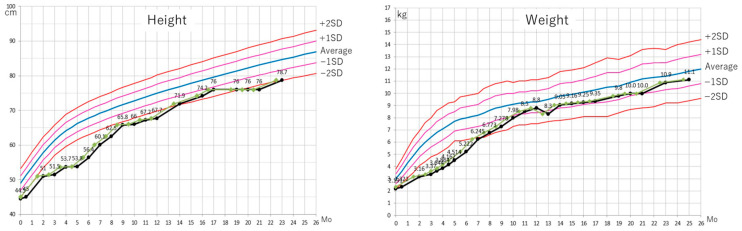
Standard Growth Curves for Boys.

Phase I was characterized by a significant amount of time dedicated to promptly puncturing newly formed blisters and applying wound dressings, thereby limiting habilitation/rehabilitation intervention time. Given that contact readily leads to blister formation, a critical balance between blister prevention and motor development was required. The growth curves are shown in Figure 1. The limbs appeared thinner than the trunk, which suggested delayed muscle development. The infant’s weight had increased to 3200 g by postnatal day 81, and the infant’s general condition improved. Physical therapy was initiated with the goals of exploring positions less vulnerable to skin peeling, promoting gross motor development, and improving oxygenation, which still required supplemental oxygen (5 L/min). In terms of gross motor skills, lateral neck rotation was acquired at three months of age.

Although Weisman’s guidelines recommend aiming for developmental motor milestone attainment [13], the fragile skin condition prevented the implementation of extensive physiotherapy interventions. Therefore, the interventions capitalized on the opportunities afforded by necessary care and the mother–infant relationship. These opportunities included: (a) finding positions to facilitate spontaneous activities and (b) minimal Range-of-Motion (ROM) exercises. The infant wore wound dressings on the head, neck, limbs, and trunk to protect the skin and promote wound healing and epithelialization. Joint mobility was particularly restricted at the elbow and knee joints, whereas gentle mobilization of the shoulders and hips was allowed. The infant’s primary static position was supine, with the neck in proper rotation and both upper limbs extended in a slightly abducted position. A towel was placed under the knees to position the hips and knees in slight flexion.

Additionally, while encouraging spontaneous movement and observing behavioral cues, active positioning was explored to avoid pressure on areas vulnerable to blister formation. Specifically, when supine, cushions were placed on the posterior surfaces of both upper limbs to allow activity along the body’s midline. For both lower limbs, cushions thicker than those used in the standard position were used to enhance flexion, thereby promoting abdominal muscle activity. Seated positioning was incorporated during blister care. The only intervention other than positioning was ROM exercise, performed at the lowest possible frequency. Owing to concerns about blister formation during exercises with the wound dressings in place, interventions were limited to confirming joint ROM twice daily, only when the dressings were removed for routine care.

On postnatal day 101, the mother was permitted to hold the child using sponge cushions and to participate in blister care with the nurses. The nurses and mother were instructed to: (1) gently round the lumbar area to ameliorate the hypertonicity of the lower back when holding the infant; (2) provide balanced auditory and visual stimulation from both sides to promote attention and visual development; and (3) provide sensory input to each finger individually when petroleum jelly was applied for skin protection, aiming to stimulate perceptual development.

The standard growth curve for the case is shown alongside that for standard Japanese boys. (A) The case followed the standard −2 S.D. for height, and (B) −1 S.D. for weight.

### 2.3. Gross Motor Training with Minimal Body Contact (Phase II: Postnatal Days 108–180)

During Phase II, positioning restrictions were gradually eased, and minimal contact was permitted for adjusting the infant’s posture during rehabilitation sessions. The infant was generally kept in a supine position. To promote epithelialization, a soft-silicone polyurethane-based wound dressing (Mepilex^®^, Mölnlycke, Gothenburg, Sweden) was applied to the head, face, limbs, hands, feet, and trunk, with particular focus on the elbow and knee joints. Owing to comorbid tracheomalacia, nasal Continuous Positive Airway Pressure (CPAP) was initiated at 148 days of age. Due to the concern for blister formation, we prioritized: (a) minimal-contact motor development activities and (b) non-physical pulmonary rehabilitation, adhering to the primary recommendations of the physiotherapy guideline [13].

Physical contact with the limbs and trunk, as well as holding the infant via the Cradle carry technique, was newly permitted for therapists. Unstable neck control was observed at five months of age. To facilitate ventilation efficacy and reduce abnormal trunk muscle tone, prone positioning began on day 156. This was performed during rehabilitation sessions under strict supervision, monitoring the infant’s behavioral cues, alongside the mother. The infant then gradually gained the ability to turn the neck bilaterally. Sucre N (Assist Co., Ltd., Osaka, Japan) was used as a positioning aid to acquire a stable sitting posture. Towels were used to facilitate spontaneous activity and further encourage trunk flexion. To facilitate forward movement of the arms, cushions were placed to support both the upper limbs.

### 2.4. Initiation of Functional Habilitation (Phase III: Postnatal Days 181–240)

Phase III marked the allowance of body contact for habilitation/rehabilitation maneuvers. On postnatal day 210, the child was transferred from the Neonatal Intensive Care Unit (NICU) to the Growing Care Unit. The wound dressings remained largely unchanged, though the hand and foot coverings began to loosen. Sitting training using Sucre N was initiated by the ward nurses. In addition, the infant was briefly placed in a right lateral decubitus position after feeding to prevent vomiting. However, ventilation support was upgraded to Synchronized Inspiratory Positive Airway Pressure (SiPAP) due to the exacerbation of tracheomalacia symptoms. The habilitation/rehabilitation strategy was modified to initiate four key areas: (a) assessment of motor development and functional issues, (b) gross motor training, (c) hands-on respiratory rehabilitation, and (d) instruction for caregivers.

With the further easing of body contact restrictions, we conducted evaluations of motor developmental delay, postural response, muscle growth, abnormal muscle tone (e.g., hypertonicity in the back), and restricted ROM, primarily in large joints. Table 1 summarizes the motor development process for this patient. Spontaneous bilateral upper limb movement along the midline gradually improved, and reaching and grasping became observable. We then initiated rolling practice by utilizing a wedge-shaped towel to facilitate positioning from a supine to a semi-lateral recumbent position, concurrently guiding reaching movements toward objects of interest. As back muscle strength improved, the child gained the ability to lift his head momentarily, in addition to exhibiting improved upper-limb activity in the prone position. The child developed a preference for sitting and was able to observe and track objects in multiple directions.

Hands-on respiratory rehabilitation was initiated. Due to impaired thoracic muscle flexibility and coordination, respiratory support training synchronized with breathing and thoracic ROM exercises was implemented while carefully avoiding blister formation. The introduction of SiPAP necessitated attention to prevent mechanical irritation to the facial skin during exercise and episodes of crying.

Psychologically, caregivers were encouraged to vary their roles to facilitate shifting the infant’s focus of interest and promote person recognition. Caregivers, including family members, were instructed to hold the infant in a prone position against their chest and perform thoracic mobilization synchronized with the infant’s breathing.

### 2.5. Habilitation for Acquisition of Mobility and Fine Motor Skills (Phase IV: Postnatal Days 241–310)

During Phase IV, specific restrictions regarding posture and habilitation/rehabilitation implementation were eliminated. Wound dressings for the face were discontinued but generally maintained for the remaining body parts. Soft protective mats were used to prevent blister formation while allowing for extended periods in the prone position. To further facilitate the development of fine motor skills, the program specifically targeted: (a) the development of trunk function and other gross motor movements, (b) the coordination of spontaneous upper limb and trunk movements, and (c) structuring the inpatient environment to incorporate simple training provided by caregivers and prevent blister formation during voluntary activity. It is noteworthy that the softness of the blister-preventive mattress limited the infant’s ability to exhibit spontaneous movements.

Body growth and motor development were continuously evaluated, and areas with residual sensory hypersensitivity and those vulnerable to blistering were identified. ROM limitation was absent at the elbow and knee joints when dressings were removed, with only mild limitation remaining at the finger joints. To promote independent sitting, weight bearing on the lower body was encouraged by gentle support for the buttocks and increasing sensory input during assisted floor sitting. In the later phase, lower limb elevation in the supine position facilitated the ability to roll.

The child began reaching out to grasp objects and bringing them to his mouth. Additionally, the movement of pushing the mother during breastfeeding also suggested increased upper-limb activity. We explored positioning and environmental structuring to encourage bimanual play in the side-lying and prone postures. Cushions were applied to facilitate manipulation of both upper limbs in front while in the lateral recumbent position or inserted under the chest to support the large head while in the prone posture.

To develop coordination between trunk and limb movements and promote abdominal muscle activity, we incorporated reaching exercises and trunk extension and rotation ROM exercises while seated. Caregivers were instructed to facilitate pelvic posterior tilt while the child was seated and during daily care, such as diaper changes. Maintaining a seated position on the bed and reaching for nearby objects with the ipsilateral arm while extending it was eventually achieved. No adverse events were observed during the rehabilitative intervention.

### 2.6. Discharge (Phase V: Postnatal Day 311)

The infant achieved stable head/neck control accompanied by bilateral rotation, upper limb elevation, and reaching movements to both sides in the supine position. Bilateral lower limb flexion and extension movements, rolling over from the supine to both lateral positions with prompting, sitting on the floor with minimal caregiver support, and head lifting in the prone position were also attained. However, crawling and standing upright with hand support remained challenging. Mild elevation of muscle tone and ROM limitations persisted in the lumbar and upper trunk regions. Additionally, state regulation became possible.

The following instructions were provided to the parents upon discharge: (1) Ensuring Sensory Input: Apply a baseline to each finger while monitoring for blister formation, and lift the patient to place the soles of the feet on the floor. While sitting, gently press the buttocks down to help the child recognize the center of gravity in the lower body. (2) Positioning: (a) In the supine position, a cushion or similar support should be placed behind the shoulder joints to maintain scapular abduction. (b) To promote voluntary abdominal muscle movement, cushions or similar items should be inserted behind the lower limbs to maintain both hips in a slightly flexed position. (c) To promote upper limb movement, the arms should be positioned in front of the face during both the side-lying and prone positions. (d) During prone positioning, cushions or similar items should be inserted from the shoulder girdle to below the chest to support the upper body. (3) Expanding ROM: Facilitate posterior pelvic tilt during holding, sitting on the lap, and diaper changes. (4) Facilitating Active, Coordinated Movement of Limbs and Trunk: Promote scapular abduction with horizontal reaching movements. Promote upper trunk extension and rotation during diagonal upward-reaching movements. (5) Development of Supine and Prone Position Movements: (a) Practice rolling over from the supine position using reaching movements. (b) In the prone position, lower body weight bearing is needed to facilitate head lifting, followed by support of the upper body with both forearms and palms.

## 3. Discussion

Dowling–Meara Epidermolysis Bullosa (EB) simplex typically manifests as widespread blister formation following minor trauma to areas vulnerable to external forces, such as the extremities and major joints of the body. The most fundamental principle of EB habilitation/rehabilitation for infants is to prevent blister formation resulting from contact-based therapy. Consequently, especially during the initial intervention phase, essential care activities such as blister management, feeding, and diaper changes were leveraged as opportunities for training aimed at developing motor and sensory functions. Table 2 summarizes the strategies employed for each phase of contact restriction.

The primary factors that hinder motor development are severe pain from blister formation, which reportedly induces reduced brain volume in the frontal and parietal regions and contributes to intrinsic behavioral issues [15,16], and malnutrition due to dysphagia caused by esophageal stricture and exudate from systemic blister formation [17,18]. Secondary factors, such as behavioral and environmental issues—including movement restraint from wound dressings, reduced sensory input via the body surface, and prolonged fixed positions—also contribute to this process [13,15,19]. Positioning restrictions can lead to Range-of-Motion (ROM) limitations, including thoracic stiffness and muscle tone elevation, which subsequently result in reduced respiratory efficiency and poor coordination between the limbs and trunk. This further leads to the underdevelopment of limb muscles, imbalanced physical growth, and bone health problems [20]. The overarching purpose of rehabilitative interventions is to treat these secondary issues [12,13].

Desensitization is one of the most basic rehabilitative approaches for infants with developmental disorders, and it is well-documented in the context of autism treatment [21]. The development of sensory processing is vital for motor, behavioral, and emotional growth [22], and affective touch is beneficial for the mental development of infants [23]. Since sensory functioning is severely affected by blister formation and dressings in infants with EB, we posit that sensory rehabilitation focusing on habituation for sensory input, desensitization of irritated skin, and sensorimotor integration is an essential approach in EB habilitation.

As the child progressed monthly, became capable of spontaneous movement, and skin fragility improved, the range of intervention methods increased. Since it remained difficult to entirely avoid skin damage during manual habilitation/rehabilitation, minimal manual training was implemented, focusing on the development of limb coordination during reaching movement, which showed a remarkable delay. Conversely, we placed a greater emphasis on behavioral cues, as represented by expressions of comfort or discomfort [24], when instructing the positioning to encourage movement and desensitize discomfort. As the infant’s response to visual input was age-appropriate, we routinely practiced gross motor skills using visual cues by sharing objects of interest with parents and caregivers.

The most significant difference between our intervention and previously reported cases is the term ‘after birth’. Although the majority of previous reports have focused on the importance of finger splinting during the most severe period [25], the current intervention was initiated immediately after birth, and there was no room for splinting the small and vulnerable fingers. Instead, each finger was independently covered by a dressing, which sufficiently extended the fingers, preventing deformities and flexion contractures. Furthermore, the ROM was preserved with the current rehabilitative intervention. Moving forward, occupational therapy interventions will likely become increasingly important for addressing issues such as fine motor development and the application of skin protection techniques and assistive devices for various problems in Activities of Daily Living (ADL) after discharge [12].

It is noteworthy that the current patient suffered from tracheomalacia and required oxygen treatment, including SiPAP [5]. Therefore, the need for respiratory rehabilitation was greater than in general cases. Secondly, this case was experienced during the last stage of the COVID-19 pandemic, and human-to-human contact, especially the involvement of parents, was partially restricted. Holding the baby is crucial not only for the child’s emotional development but also for maternal bonding, and a prolonged ICU stay immediately after birth was a risk factor for weakening the bond with the mother and other family members [26]. Although maximum permission was given to the mother to be involved in the care and to have contact with the infant, concerns remain about the emotional development. On the other hand, from a broader habilitation/rehabilitation standpoint, the strict prioritization and selection of the intervention menu in the current case will be informative for various limited-resource situations, such as those in countries with limited capacity.

The current report has some limitations. Existing guidelines propose the importance of determining objective developmental milestones in comparison to healthy infants, which serves as an aim for implementing habilitation intervention [13]. While we reported the gross motor developmental milestones as observed in Table 2, the position–activity management and rehabilitative treatment of the current case were completely dominated by systemic and dermatological conditions dictated by medical requirements. Suggestions for modifying infant management and habilitation from the standpoint of developmental milestones will become possible upon the accumulation of such clinical reports or studies. In addition, the present report may lack generalizability due to the rarity and variability of EB and the comorbid tracheomalacia requiring the oxygen treatments. We hope that this report will contribute to the clinical advances of EB and that further reports will enable comparisons and further clinical development.

## 4. Conclusions

In the habilitation/rehabilitation of newborns and infants with Epidermolysis Bullosa (EB) simplex, physical contact with the child is highly restricted. It is essential to integrate as many activities as possible into training opportunities, including medical procedures, routine care, and communication with medical professionals and parents of children with disabilities. The current article is the first to report the importance of sensory training and behavioral cue application as a common approach for EB habilitation/rehabilitation. It also demonstrates a specific feature of respiratory rehabilitation for an infant with the most severe dermatological disorder.

## Figures and Tables

**Table 1:** Motor development process: standard months vs. the case.

Normal Motor Development	Standard	Case
Head rotation in supine	1 Mo	3 Mo
Brief head lift in prone		6 Mo
Grasping objects placed in the hand	4 Mo	6 Mo
Supporting head on elbows in prone	5 Mo	7 Mo
Sitting with hands supporting the body for several seconds	6 Mo	9 Mo
Grasping feet in supine		10 Mo
Reaching out to grasp objects		6 Mo
Supporting body weight with one hand in prone (and reaching with the other)	7 Mo	9 Mo
Reaching for objects to the side while seated	8 Mo	9 Mo
Visual tracking and person recognition		7 Mo
Maintaining seated position		10 Mo

**Table 2:** Summary of the Habilitation/Rehabilitation Strategy by Phase.

Phase (Postnatal Days)	Developmental/Clinical Issues	Restriction Status	Habilitation/Rehabilitation Strategy
I (81–107)	Mobility restricted by wound dressings (elbow, knee). Limb growth delay. Constant supine posture. Hypertonicity in the lower back.	Habilitation/Rehabilitation contact allowed.	(a) Positioning: To facilitate spontaneous activities. (b) Minimal Range-of-Motion (ROM) exercises.
II (108–180)	Developmental delay: Partial neck control. Exacerbation of tracheomalacia requiring CPAP administration.	Positioning restrictions gradually eased.	(a) Minimal-contact motor development procedures (including sitting training). (b) Non-physical pulmonary rehabilitation.
III (181–240)	Developmental delay: Inability to lift head in prone, limited object grasp/visual tracking. Impaired thoracic muscle flexibility/coordination. Exacerbation of tracheomalacia requiring SiPAP administration.	Any training with contact allowed (with minimal amount).	(a) Initial assessment of motor development and functional issues. (b) Gross motor training. (c) Hands-on respiratory rehabilitation. (d) Focused instruction for caregivers (e.g., assisted sitting position).
IV (241–310)	Developmental delay in gross and fine motor skills. Insufficient upper limb and trunk coordination.	Specific restrictions eliminated.	(a) Gross motor training. (b) Trunk–upper limb coordination training. (c) Further structuring of the inpatient living environment.
V (311) (Discharge)	Developmental delay: Partial sitting achieved; crawling and standing not acquired. Mild hypertonicity and mild ROM restriction in the trunk.	Home Discharge.	Caregiver Instruction: Ensure sensory input (fingers, buttocks, plantar surface); recognize center of gravity; positioning to facilitate upper limb and abdominal muscle movements; prone posture; trunk ROM with reaching training; gross motor development.

## Data Availability

Data is unavailable due to privacy.

## Abbreviations

The following abbreviations are used in this manuscript:

COVID-19	Coronavirus Disease-19
CPAP	Continuous Positive Airway Pressure
EB	Epidermolysis bullosa EB
NICU	Neonatal Intensive Care Unit
ROM	Range of Motion
SiPAP	Synchronized Inspiratory Positive Airway Pressure
